# Effects of Electrolyte on Laser-Induced Periodic Surface Structures with Picosecond Laser Pulses

**DOI:** 10.3390/nano11020327

**Published:** 2021-01-27

**Authors:** Shuhei Kodama, Wataru Natsu

**Affiliations:** Department of Mechanical Engineering, Tokyo University of Agriculture and Technology, 2-24-16 Nakacho, Koganei, Tokyo 184-8588, Japan; summer@cc.tuat.ac.jp

**Keywords:** short-pulsed laser, laser-induced periodic surface structures (LIPSSs), electrochemical machining (ECM), electrolyte, nanostructure formation

## Abstract

Short-pulsed laser-induced periodic surface structures (SPLIPSSs) have the possibility to control tribology, wettability and biocompatibility. Nevertheless, the optimal structure depends on each functionality, which has not been clarified. The hybrid process with a short-pulsed laser and electrochemical machining (SPLECM) is, then, proposed to fabricate micro/nano hybrid structures and to modify the surface composition for providing high functionalities with material surfaces. Electrochemical machining is a well-established micro-elution and deposition method with noncontact between a workpiece and a tool. In this study, the effects of electrolytes on SPLIPSSs were investigated experimentally by the picosecond laser irradiation on 304 stainless steel substrates in various electrolytes. The geometry of SPLIPSSs depended on the types and the concentration of electrolytes. In the case of copper nitrate solution and copper sulfate solution, LIPSSs and spheroidization of copper were obtained. This study demonstrated the possibility of SPLECM to fabricate micro/nano structures and to control surface composition.

## 1. Introduction

Micro/Nanostructures can alter tribology [1,2], wettability [3,4], optical properties [5,6] and bioaffinity [7,8] on the material surface. The short-pulsed laser (SPL) is an appropriate method to fabricate nanostructures, called laser-induced periodic surface structures (LIPSSs), inducing a reduction of friction [9,10], water repellency and hydrophilicity [11,12,13], anti-reflection [14,15] and improvement of biocompatibility [16,17]. LIPSSs with a periodicity of 0.5–0.85 times the laser wavelength are fabricated through the self-organizing way [18]. The plasma waves, induced via protrusions on a surface based on the parametric decay [18,19,20,21], induced surface plasmons by the interference with the incident light, resulting in periodic Coulomb explosions [22] and ablation. Nevertheless, the appropriate structure for each functionality has not been clarified, although it has been reported that fine structures are effective to alter the surface reaction. In addition, fabrication of multiscale structures has been recently required to provide high added value with materials.

The hybrid manufacturing process is then proposed that combines an SPL and electrochemical machining (ECM), a noncontact machining method with electrolytic elution and deposition [23,24], to fabricate multiscale structures effectively and to control surface composition, in contrast to the laser-assisted electrochemical machining that combines a long-pulsed laser and ECM to increase the efficiency of ECM by enhancing thermal electrochemical action and removing the passive film [25,26,27,28,29,30].

The objectives of this study were to verify the effects of electrolytes on the fabrication of LIPSSs since a few studies have reported that LIPSS geometry depends on the irradiation environment [31]. We also aimed to spheroidize copper since ECM can coat with copper using copper nitrate (Cu(NO_3_)_2_) solution and copper sulfate (CuSO_4_) solution [32,33,34,35] since a long-pulsed laser or a continuous laser have been mainly used for laser enhanced electroless plating and the effects of an SPL on metal deposition with the fabrication of LIPSSs have never been studied. Experiments of laser irradiation with 1064 nm and 20 ps laser pulses at 0.04–1.0 J/cm^2^ on the workpiece of 304 stainless steel under air, water, sodium chloride (NaCl) solution, sodium nitrate (NaNO_3_) solution, copper nitrate (Cu(NO_3_)_2_) solution and copper sulfate (CuSO_4_) solution, were conducted to fabricate LIPSSs in liquids and to change the surface composition. The spot size was 260 µm. In liquids, LIPSSs with less periodicity than LIPSSs under air were fabricated, and spheroidization of copper was obtained in Cu(NO_3_)_2_ solution and CuSO_4_ solution. This paper describes the formation of LIPSSs in various electrolytes and the effects of short-pulsed laser irradiation in electrolytes on the surface composition.

## 2. Experiment

A 304 stainless steel plate (10 mm × 10 mm × 1.5 mm thickness) was used as a workpiece for this study to fabricate LIPSSs in various liquids. As liquids, we prepared water, 5 and 10 wt% NaCl solutions, 5 and 10 wt% NaNO_3_ solutions, 5 and 10 wt% Cu(NO_3_)_2_ solutions and 5 and 10 wt% CuSO_4_ solutions. NaCl solution and NaNO_3_ solution are often used for ECM [23,24,36,37], and Cu(NO_3_)_2_ solution and CuSO_4_ solution are used for electrochemical deposition of copper [32,33,34,35].

Short-pulsed laser irradiation experiments on a 304 stainless steel plate under air, water and electrolytes were conducted with a picosecond-pulse laser oscillator (EKXPLA, PL 2250-50P20) with 20 ps pulse duration. A longer pulse duration laser has a lower cost and more stable laser irradiation, and 20 ps is the approximate maximum of the collisional relaxation time of metals which is a key for the fabrication of LIPSSs.

**Figure 1:**
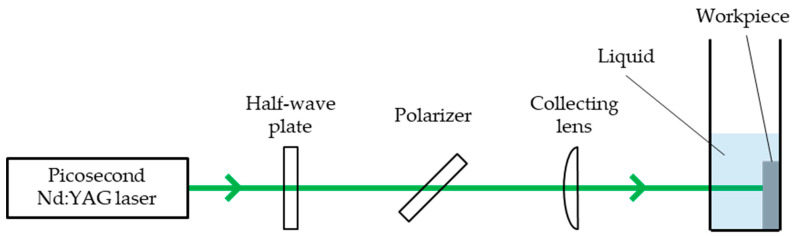
Schematic diagram of the experimental setup for laser irradiation on the material surface set vertically to the ground.

Figure 1 shows the schematic diagram of the experimental setup including a half-wave plate to change the polarization state and to adjust laser power, a polarizer to isolate the specific polarization of light and a collecting lens with a focusing range of 150 mm. The workpiece was set in a quartz cell and set vertically to the ground. A workpiece was irradiated by a Gaussian laser beam on the fixed point without scanning. A picosecond Nd:YAG laser with a pulse duration of 20 ps, a wavelength of 1064 nm and a frequency of 50 Hz was used. The number of pulses *N* was set to 1–1000. The laser fluence *F* was set to 0.04–1.00 J/cm^2^ by using a pair of a half-wave plate and a polarizer. The entire laser power was measured via the laser power meter, and the accurate beam profile was calculated by calibrating the measured beam profile via a charge-coupled device (CCD) beam profiler.

The surface morphology of the central irradiated area was observed using a scanning electron microscope (SEM, XL30, Philips, Eindhoven, The Netherlands). The surface geometries were further analyzed using a laser microscope (VK-X100, KEYENCE, Osaka, Japan) and an atomic force microscope (AFM, MultiMode 8, Bruker AXS, Karlsruhe, Germany). The periodicity of the LIPSSs was evaluated by a two-dimensional Fourier transform performed on the SEM image along the polarization direction. The elemental analysis was conducted by the energy dispersive X-ray spectroscopy (EDX, EDAX DX-4, Philips, Eindhoven, The Netherlands).

## 3. Results and Discussion

**Figure 2:**
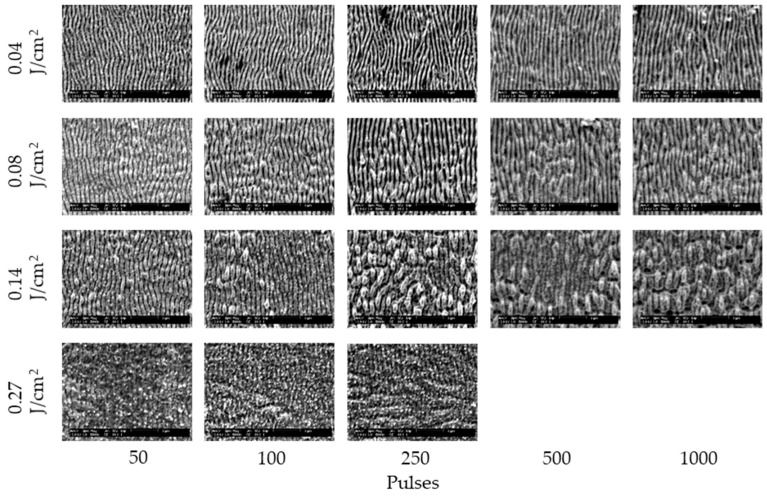
SEM images of 304 stainless steel irradiated with *N* = 50–1000 pulses at *F* = 0.04–0.27 J/cm^2^ under air. The laser beam has the polarization in a lateral direction.

**Figure 3:**
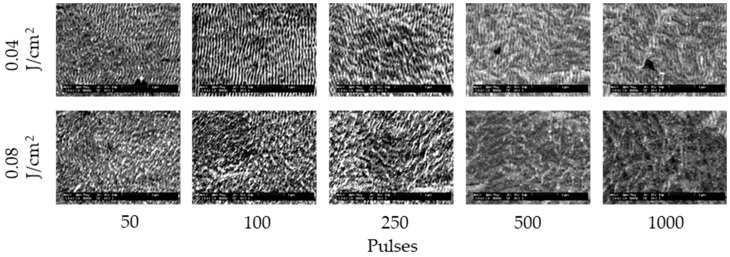
SEM images of 304 stainless steel irradiated with *N* = 50–1000 pulses at *F* = 0.04–0.08 J/cm^2^ in water. The laser beam has the polarization in a lateral direction.

**Figure 4:**
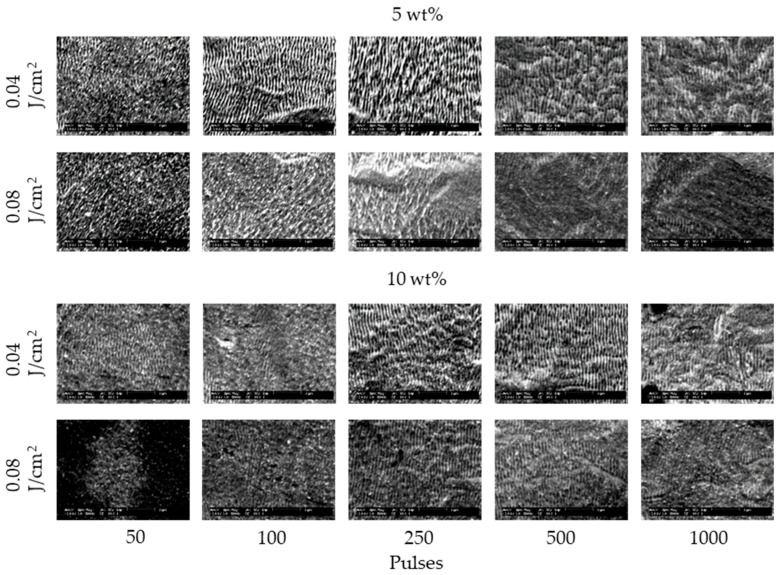
SEM images of 304 stainless steel irradiated with *N* = 50–1000 pulses at *F* = 0.04–0.08 J/cm^2^ in 5 and 10 wt% NaCl solutions. The laser beam has the polarization in a lateral direction.

**Figure 5:**
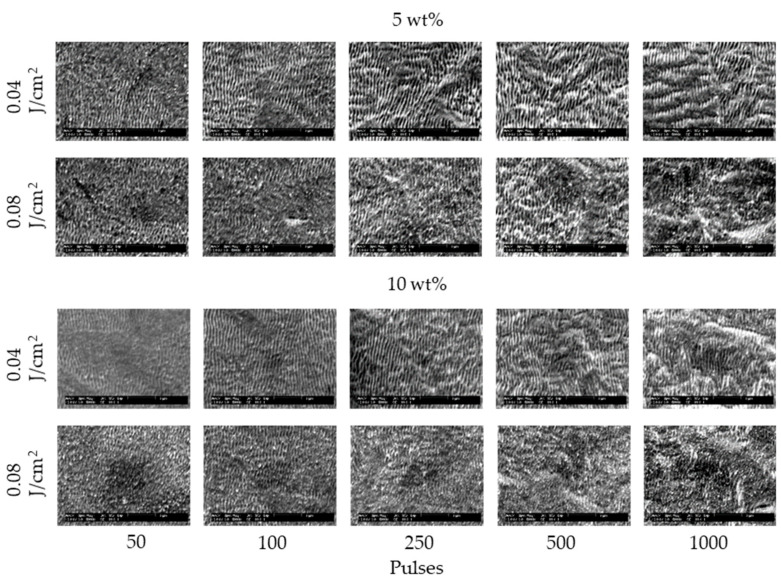
SEM images of 304 stainless steel irradiated with *N* = 50–1000 pulses at *F* = 0.04–0.08 J/cm^2^ in 5 and 10 wt% NaNO_3_ solutions. The laser beam has the polarization in a lateral direction.

**Figure 6:**
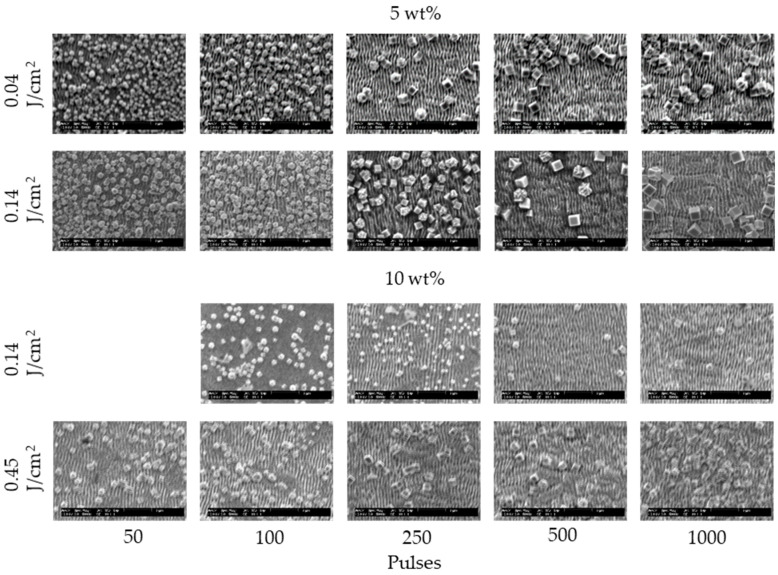
SEM images of 304 stainless steel irradiated with *N* = 50–1000 pulses at *F* = 0.04–0.45 J/cm^2^ in 5 and 10 wt% Cu(NO_3_)_2_ solutions. The laser beam has the polarization in a lateral direction.

**Figure 7:**
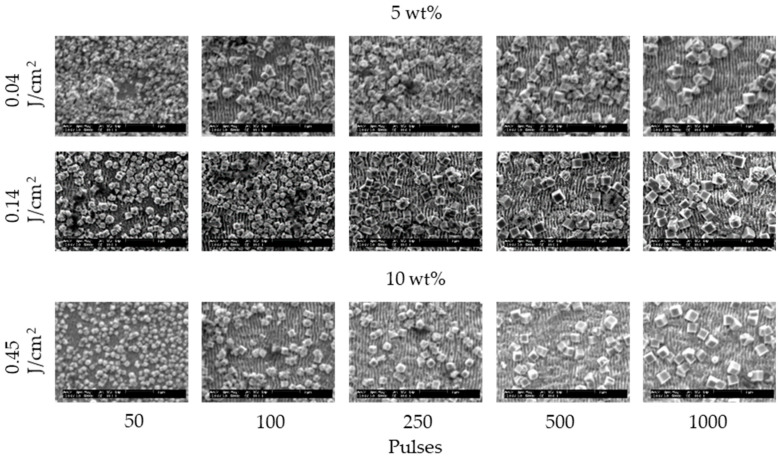
SEM images of 304 stainless steel irradiated with *N* = 50–1000 pulses at *F* = 0.04–0.45 J/cm^2^ in 5 and 10 wt% CuSO_4_ solutions. The laser beam has the polarization in a lateral direction.

Figure 2 and Figure 3 show the SEM images of 304 stainless steel irradiated at *F* = 0.04–0.27 J/cm^2^ for *N* = 50–1000 pulses under air and water, respectively. These demonstrate that LIPSSs perpendicular to the laser polarization were fabricated on the irradiated surface under air and water; on the other hand, LIPSSs with lower periodicity were fabricated in water when the low laser fluence compared to LIPSSs fabricated under air since large ablation on the irradiated surface causes disappearance of LIPSSs due to suppression of plasma expansion by water [38,39,40,41]. Figure 4 and Figure 5 show the SEM images of 304 stainless steel irradiated at *F* = 0.04–0.08 J/cm^2^ for *N* = 50–1000 pulses in 5 and 10 wt% NaCl solutions and 5 and 10 wt% NaNO_3_ solutions, respectively. These demonstrate that LIPSSs with lower periodicity were fabricated at lower laser fluence in both solutions than LIPSSs fabricated under air, similar to LIPSSs fabricated in water. Figure 6 and Figure 7 show the SEM images of 304 stainless steel irradiated at *F* = 0.04–0.45 J/cm^2^ for *N* = 50–1000 pulses in 5 and 10 wt% Cu(NO_3_)_2_ solutions and 5 and 10 wt% CuSO_4_ solutions, respectively. These demonstrate that LIPSSs with lower periodicity were fabricated at a wide-range laser fluence in both solutions, and copper particles were deposited on the irradiated surface and grew with increasing the number of pulses since plasma generation reduced the copper ion in the solutions to form copper particles on the irradiated surface and made these grow [42,43,44].

**Figure 8:**
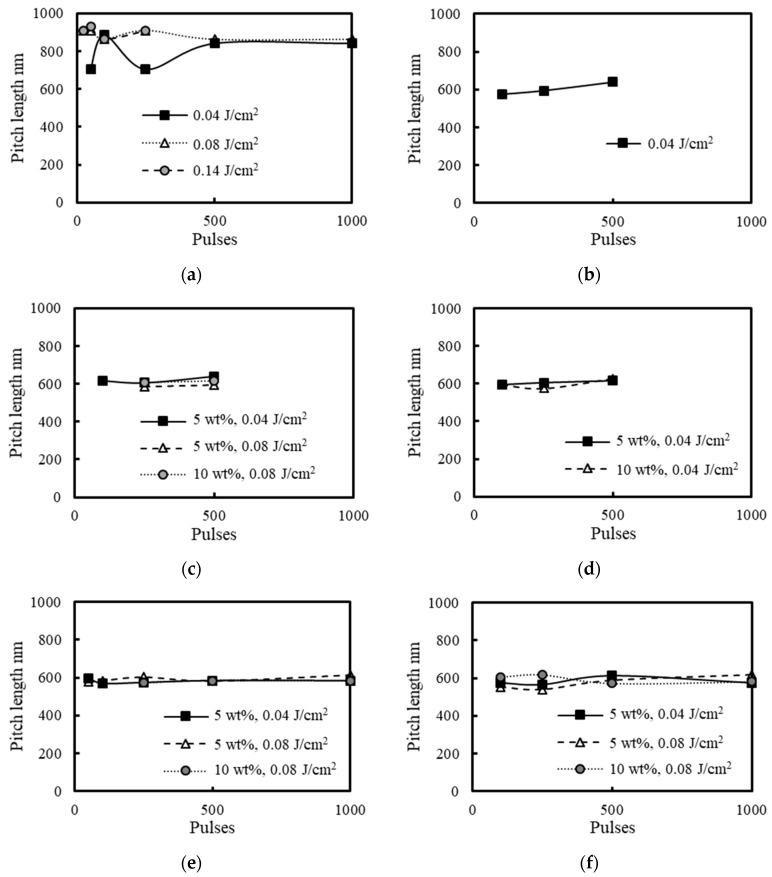
Relationship between the periodicity of laser-induced periodic surface structures (LIPSSs) and laser irradiation conditions under (**a**) air, (**b**) water, (**c**) 5 and 10 wt% NaCl solutions, (**d**) 5 and 10 wt% NaNO_3_ solutions, (**e**) 5 and 10 wt% Cu(NO_3_)_2_ solutions and (**f**) 5 and 10 wt% CuSO_4_ solutions.

Figure 8 shows changes in the periodicity of LIPSSs fabricated on 304 stainless steel surfaces under air, water, 5 and 10 wt% NaCl solutions, 5 and 10 wt% NaNO_3_ solutions, 5 and 10 wt% Cu(NO_3_)_2_ solutions and 5 and 10 wt% CuSO_4_ solutions for each irradiation condition. The periodicity of LIPSSs on the 304 stainless steel surfaces under air was about 700–900 nm, which was about 0.65–0.85 times the laser wavelength. This phenomenon was attributed to the surface plasma waves whose wavelength is 0.50–0.85 times the laser wavelength, and the increase of the electron density extends it, explained by the parametric decay [18,19,20,21]. On the other hand, the periodicity of LIPSSs on the 304 stainless steel surfaces in water, 5 and 10 wt% NaCl solutions, 5 and 10 wt% NaNO_3_ solutions, 5 and 10 wt% Cu(NO_3_)_2_ solutions and 5 and 10 wt% CuSO_4_ solutions was about 600 nm, which was about 0.56 times the laser wavelength since the refractive index of water is 1.33 [45], shortening the laser wavelength and the wavelength of the surface plasma waves. In the solutions, the periodicity is approximately the same since the refractive index of 5 and 10 wt% NaCl solutions, 5 and 10 wt% NaNO_3_ solutions, 5 and 10 wt% Cu(NO_3_)_2_ solutions and 5 and 10 wt% CuSO_4_ solutions is close to the refractive index of water [46,47,48,49,50].

**Figure 9:**
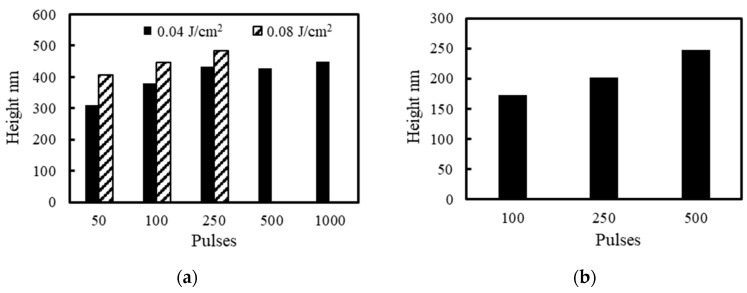

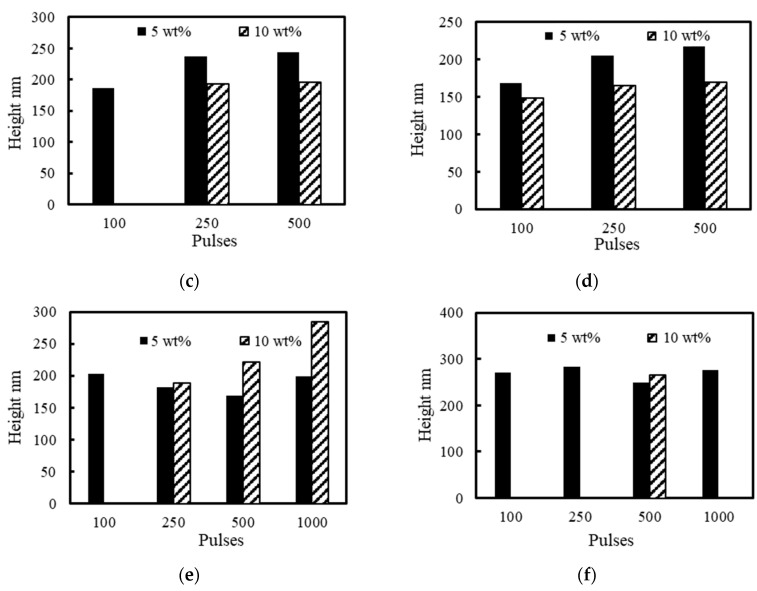
Relationship between the height of LIPSSs and laser irradiation conditions under (**a**) air, (**b**) water, (**c**) 5 and 10 wt% NaCl solutions, (**d**) 5 and 10 wt% NaNO_3_ solutions, (**e**) 5 and 10 wt% Cu(NO_3_)_2_ solutions and (**f**) 5 and 10 wt% CuSO_4_ solutions.

Figure 9 shows the changes in height of LIPSSs fabricated on 304 stainless steel surfaces under air, water, 5 and 10 wt% NaCl solutions, 5 and 10 wt% NaNO_3_ solutions, 5 and 10 wt% Cu(NO_3_)_2_ solutions and 5 and 10 wt% CuSO_4_ solutions for each irradiation condition. The LIPSS height in solutions was 150–300 nm, which was lower than the LIPSS height of 300–500 nm under air [51]. This can be attributed to the attenuation of light in solutions decreasing the intensity of the plasma waves and the suppression of plasma expansion removing LIPSSs [38,39,40,41]. With the increase of the number of pulses and fluence, the height of LIPSSs increased gradually under air, water, NaCl solutions and NaNO3 solutions, however, the height of LIPSSs in Cu(NO_3_)_2_ solution and CuSO_4_ solution was not proportional to the number of pulses since deposited copper particles which grew and were removed with the increasing number of pulses changed induction and propagation of the plasma waves. In solutions, the height of LIPSSs in Cu(NO_3_)_2_ solution and CuSO_4_ solution was larger than that in other solutions since the deposited copper particles facilitate the induction and propagation of the plasma waves, increasing the electric field intensity on the surface.

**Figure 10:**
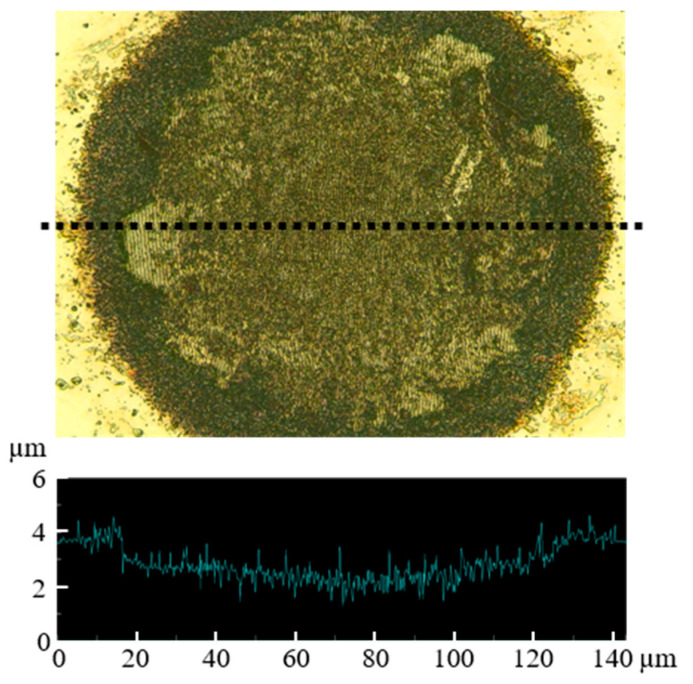
Topography of craters with *N* = 500 pulses at *F* = 0.04 J/cm^2^ under air.

**Figure 11:**
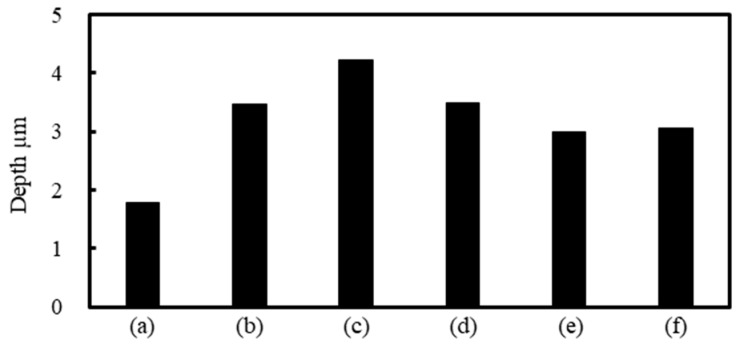
Relationship between the depth of craters and laser irradiation conditions with *N* = 500 pulses at *F* = 0.04 J/cm^2^ under (**a**) air, (**b**) water, (**c**) 5 wt% NaCl solution, (**d**) 5 wt% NaNO_3_ solution, (**e**) 5 wt% Cu(NO_3_)_2_ solution and (**f**) 5 wt% CuSO_4_ solution.

The depth of craters was measured by a laser microscope as shown in Figure 10. Figure 11 shows the depth of craters on 304 stainless steel surfaces with *N* = 500 pulses at *F* = 0.04 J/cm^2^ under air, water, 5 wt% NaCl solution, 5 wt% NaNO_3_ solution, 5 wt% Cu(NO_3_)_2_ solution and 5 wt% CuSO_4_ solution. The depth of craters in the solutions was larger than that under air since solutions suppressed the plasma expansion and induced large ablation [38,39,40,41]. In solutions, the depth of craters in NaCl solution was larger than that in other solutions due to low attenuation [25].

**Figure 12:**
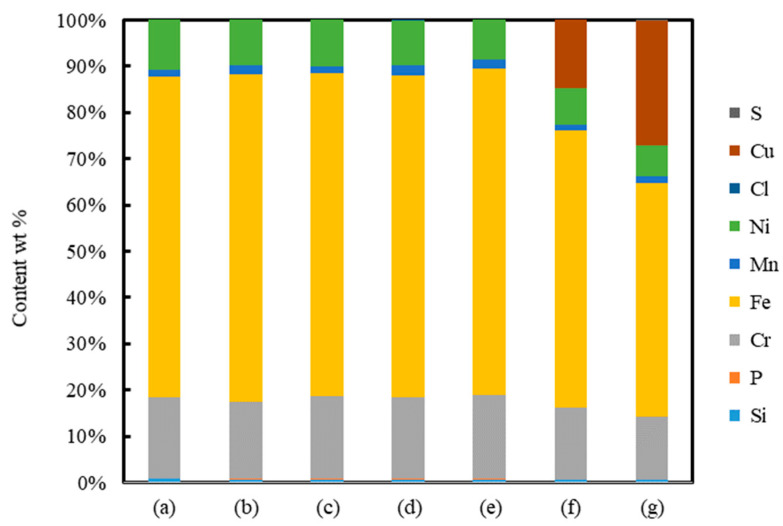
Relationship between elements of surfaces of (**a**) non-irradiated area and irradiated areas with *N* = 500 pulses at *F* = 0.04 J/cm^2^ under (**b**) air, (**c**) water, (**d**) 5 wt% NaCl solution, (**e**) 5 wt% NaNO_3_ solution, (**f**) 5 wt% Cu(NO_3_)_2_ solution and (**g**) 5 wt% CuSO_4_ solution.

Figure 12 shows the elements of surfaces of non-irradiated and irradiated areas with *N* = 500 pulses at *F* = 0.04 J/cm^2^ under air, water, 5 wt% NaCl solution, 5 wt% NaNO_3_ solution, 5 wt% Cu(NO_3_)_2_ solution and 5 wt% CuSO_4_ solution. The elements of surfaces were almost the same under air, water, NaCl solution and NaNO_3_ solution; on the other hand, the surfaces in Cu(NO_3_)_2_ solution and CuSO_4_ solution had the copper element, meaning that copper particles were deposited on the surfaces by laser irradiation in Cu(NO_3_)_2_ solution and CuSO_4_ solution [42,43,44]. The composition of copper deposition of CuSO_4_ was larger than Cu(NO_3_)_2_. It is considered that the lower attenuation of the laser causes more copper deposition in CuSO_4_ solution due to Cu(NO_3_)_2_ of a larger molecular weight scattering a laser beam, leading to high LIPSSs and deep craters in CuSO_4_ solution.

## 4. Conclusions

LIPSSs were fabricated on the 304 stainless steel surface by using a 20 ps laser in water, NaCl solution, NaNO_3_ solution, Cu(NO_3_)_2_ solution and CuSO_4_ solution at low fluence. In the case of Cu(NO_3_)_2_ solution and CuSO_4_ solution, copper particles were deposited on the irradiated surfaces analyzed by EDX. The periodicity of LIPSSs in solutions was about 600 nm, which was shorter than that under air due to the refractive index shortening the laser wavelength. The LIPSS height of 150–300 nm in solutions was lower than that under air due to the attenuation of light; on the other hand, the depth of craters in the solutions was larger than that under air due to the suppression of plasma expansion and large ablation. The 20 ps laser irradiation in solutions can fabricate LIPSSs with shorter periodicity, depositing copper particles.
